# Quality of delivery of “right@home”: Implementation evaluation of an Australian sustained nurse home visiting intervention to improve parenting and the home learning environment

**DOI:** 10.1371/journal.pone.0215371

**Published:** 2019-05-06

**Authors:** Lynn Kemp, Tracey Bruce, Emma L. Elcombe, Teresa Anderson, Graham Vimpani, Anna Price, Charlene Smith, Sharon Goldfeld

**Affiliations:** 1 School of Nursing and Midwifery, Western Sydney University, Ingham Institute for Applied Medical Research, Liverpool, New South Wales, Australia; 2 Sydney Local Health District, Camperdown, New South Wales, Australia; 3 Community Child and Family Health, University of Newcastle, Regional and Rural Health Services, Hunter New England Local Health District, New Lambton, New South Wales, Australia; 4 Centre for Community Child Health, The Royal Children’s Hospital, Parkville, Victoria, Australia; 5 Population Health, Murdoch Children’s Research Institute, Parkville, Victoria, Australia; 6 Department of Paediatrics, University of Melbourne, Parkville, Victoria, Australia; 7 Australian Research Alliance for Children and Youth, Canberra City, Australian Capital Territory, Australia; Monash University, AUSTRALIA

## Abstract

**Background:**

Home visiting programs are implemented in high income countries to improve outcomes for families with young children. Significant resources are invested in such programs and high quality evaluations are important. In the context of research trials, implementation quality is often poorly reported and, when reported, is variable. This paper presents the quality of implementation of the right@home program, a sustained nurse home visiting intervention trialled in Australia, and delivered in a ‘real world’ context through usual child and family health services. right@home is structured around the core Maternal Early Childhood Sustained Home-visiting (MECSH) program, which is a salutogenic, child focused prevention model.

**Method:**

At each visit right@home practitioners completed a checklist detailing the client unique identifier, date of contact and activities undertaken. These checklists were collated to provide data on intervention dose, retention to program completion at child age 2 years, and visit content, which were compared with the program schedule. Quality of family-provider relationship was measured using the Session Rating Scale. Exploratory factor analysis was conducted to identify clusters of activities and allow qualitative assessment of concordance between program aims and program delivery.

**Results:**

Of 363 intervention families offered the program, 352 (97·0%) commenced the program and 304 (87·3%) completed the program to child age 2 years. 253 of 352 (71·9%) families who commenced the program received more than 75 percent of scheduled visits including at least one antenatal visit. Families rated the participant-practitioner relationship highly (mean 39.4/40). The factor analysis identified six antenatal and six postnatal components which were concordant with the program aims.

**Conclusions:**

The right@home program was delivered with higher adherence to program dose, schedule and content, and retention than usually reported in other home visiting research. Program compliance may have resulted from program design (visit schedule, dose, content and delivery flexibility) that was consistent with family aims.

## Introduction

The past two decades have seen increasing research evidence that sustained nurse home visiting (SNHV) can improve health and development outcomes for children and families experiencing adversity [1, 2, 3]. As a result, in high income countries like the USA [1], UK and Australia, home visiting programs are increasingly being implemented for expectant families and those with young children identified with vulnerabilities or risks for poorer outcomes. These programs are supported by policy and funding initiatives such as the US Maternal, Infant, and Early Childhood Home Visiting Program [1], which require that the implemented programs be evidence-based.

There is, however, considerable variation in the quality of programs once they are delivered in the ‘real world’ as opposed to university research-based settings, with lack of attention to defining the elements of effective service delivery processes and measurement of program fidelity [4–7]. ‘Fidelity may be defined as the extent to which delivery of an intervention adheres to the protocol or program model originally developed’ (see page 315 in [8]). Reporting of home visiting programs requires specific attention given to the number of visits families receive (retention and dose), what families receive (content), the mechanism of family engagement (professional-family relationship) [9], and the way in which these factors are monitored [6, 10].

Studies in both research and real world environments have found that the retention rates and numbers of visits can fall below program ideals and expectations [11, 12]. Recent research trials of the US Nurse Family Partnership model in England and Germany have reported low rates of retention to program completion at child-age 2-years. In the Pro Kind German study 61.8% (268 of 434 enrolled) completed the program [11]. The Building Blocks trial in England reported 76.1% program completion for those who enrolled in the Family Nurse Partnership (FNP) program, and 67.7% for all families allocated to the trial intervention group. Only 57.7%, 53.0% and 43.6% of families in the Building Blocks trial met the targets of receiving 80% of the scheduled 14 visits in pregnancy, 65% of the 28 scheduled visits in infancy, and 60% of the 22 scheduled visits during the toddler stage respectively, which they note was better than the original US-based trials [12].

In non-research implementation, reported retention and visit delivery elements of fidelity are even lower. Evaluation of the USA implementation of 35 various home visiting programs showed that retention at 12 months ranged from 3.9 to 73.0%, the proportion of families receiving the full dosage ranged from 5.3 to 26.4%, and the proportion receiving 80% of dosage ranged from 41.2 to 51.6% [13]. A study of five program models implemented nationally in the US found that only 53% of families were retained until the child was aged 12 months and less than 20% of enrolled families received the recommended number of visits [14, 15]. An evaluation of the CAPEDP Project in France [Compe´tences Parentales et Attachement dans la Petite Enfance: Diminution des Risques Lie´s aux Troubles de Sante´ Mentale et Promotion de la Re´silience] also reported that families received only 53.2% of the programmed visits [16]. Earlier evaluations found that no matter what home visiting model was implemented, families typically receive about half of the models’ intended dose [17].

As well as the retention in the program and delivering the expected number of visits, elements of fidelity include the nature of the relationship between the practitioner and parents, and delivery of program content. Effective home visiting services are relationship based, and depend on a positive relationship between the practitioner and parent/s in order to facilitate change [9, 10, 18]. A strong relationship between the practitioner and family is correlated with client service satisfaction, and supports program retention and engagement [11, 19, 20]. Further, to be effective, programs must deliver the expected program content [10]. Home visiting program content should be aligned with not only the desired program outcomes, but also address the goals and problems that matter to the family [3, 7, 9, 10]. It is uncommon for specifics of program content to be published and disclosed detail usually reports expected content rather than content actually delivered [10], although more recently greater detail is being provided. For example, the Building Blocks trial reported families generally received the expected content focus as a proportion of visiting time, however, details of specific content were not provided [12]. In a rare assessment of the consistency between the intended intervention content and that provided, an evaluation of fidelity of the CAPEDP Project in France observed both omitted and added content in delivery [16].

Research detailing the delivery of home visiting programs in real world settings is needed to unpack the “black box” (see page 217 in [21]) and understand the way that home visiting programs actually work to achieve child and family outcomes [17, 22]. To date most studies report the theoretical model: what the program is expected to deliver and how the program is expected to work, rather than actual implementation [5, 10]. Implementation research is critical to having a “well-articulated theory of change that links specific aspects of a program’s content, duration, dosage, or service delivery method to specific outcomes” (see page 3 in [23]). Frequently lacking from the literature, which focuses on whether a program achieved its intended outcomes or not, is research exploring what was actually delivered to achieve the published outcomes.

Within the context of a large, multisite, randomized controlled trial (RCT) of SNHV, the right@home trial [24], a detailed study of the intervention fidelity was undertaken, that is, retention, dose, content and relationship-base of the intervention program. We aimed to assess the implementation of the right@home program through answering three research questions: What was the retention, dose, and adherence to program schedule, in delivery of the right@home sustained nurse home visiting program?, What was the content delivered when providing the right@home program?, and Were the foci of delivered program content concordant with the program aims?

## Methods

### Study design and setting

The right@home program is a SNHV intervention that was delivered to families from the antenatal period until their child turned two years old. The right@home program was trialled in seven localities in the Australian states of Victoria and Tasmania [24]. Notably, the program was delivered within the real world setting of universal child and family health nursing services [23]. The program has demonstrated effectiveness in improving parental care of the child, responsivity, and the home learning environment for families experiencing adversity [25].

The right@home program was structured around the core Maternal Early Childhood Sustained Home-visiting (MECSH) model [26], with scheduled hourly visits of varying frequency, and incorporating the core MECSH focus modules (Learning to Communicate child development parent education program [27], social support promotion including group activities) [28] and additional focus modules (SmallTalk—a language development education program for parents of children aged 12–47 months [29], Promoting First Relationships including use of video feedback [30], healthy eating guidelines [31], infant sleeping (Infant Sleep Visit Record) [32], safety audit (Kidsafe) [33]) to improve outcomes for parent care, parent responsivity and the home learning environment [24]. The program was delivered by trained nurses (baccalaureate with postgraduate training in child and family health) supported by a baccalaureate trained social care practitioner (social worker). The social care practitioner provided instrumental and psychosocial support for families, and assisted nurses and families to leverage community resources. Each nurse supported 25–30 families per full time equivalent (FTE), with one social care practitioner supporting four FTE nurses. Each locality team of 4.0FTE nurses and 1.0FTE social worker were supervised by a team leader, providing group reflective practice supervision and case review at least monthly. right@home was designed to be delivered primarily through face-to-face visiting, however, flexibility within the program allowed for a small proportion of visits to be delivered as therapeutic telephone calls when requested by families, and group visits.

Nurses and social care practitioners were guided by a strengths-based approach and joint goal setting, based in the Family Partnership Model [18], which draws on helper and construct theory, and motivational interviewing techniques. Using the MECSH model principles, the practitioners used a salutogenic (health creating) child-focused prevention approach, supporting identified families with young children to adapt and self-manage in their parenting journey, and source the resources to parent effectively despite the difficulties and challenges they face in their day to day lives. The program aimed to improve parental care of the child, responsivity and the home learning environment through delivery of a home visiting program that supports and enables:

- positive transition to parenting;
- mother, child and family health, development and wellbeing;
- maternal–infant bonding and attachment;
- positive parenting skills;
- mothers to be future orientated and aspirational for themselves, their child and family;
- mothers and families to enhance their coping and problem-solving skills, and ability to mobilize resources;
- supportive relationships in their family and community; and
- additional support in response to family needs [9, 24, 28].

Program implementation support, training, and fidelity monitoring was conducted by the MECSH Support Service at Western Sydney University in Liverpool NSW using the standard processes for the implementation of MECSH-based licenced programs [34] internationally, with the addition of specific training in, and monitoring of the right@home focus modules. As per standard processes, program delivery was monitored quarterly, including assessment of training completion, supervision processes, dose, retention rates, content monitoring, family rating of the service, and provision of standardized feedback to each participating site. The program has an aspirational retention, dose and content performance indicator that 100% of families should receive 100% of visits and schedule content. Overall program fidelity was deemed to be satisfied if more than 75% of families received more than 75% of the visits, including at least one antenatal visit (see page 12 in [24]).

### Participants

The RCT recruited 722 pregnant women. Eligible participants were pregnant women attending the antenatal clinics in the trial areas from May 2013 to August 2014, with an expected due date before 1 October 2014. Participants needed to be less than 37 weeks gestation, have sufficient English proficiency to verbally answer interview questions, reside within the study travel boundaries, and have two or more of ten sociodemographic risk factors for adverse parent and/or child outcomes identified by risk factor screening [35, 36].

The intervention arm of the right@home trial recruited 363 families, and 352 women commenced the intervention. Table 1 shows the risk profile for all participants, those who received as least one intervention visit (right@home program recipients), and those who completed the right@home program to child age 2 years.

**Table 1:** Risk profile of right@home program participants [24].

Antenatal screening risk factor	All intervention arm participants N = 363	Program recipients N = 352	Completed program N = 304
	n (%*)	n (%*)	n (%*)
Young pregnancy (Age < 23 years)	92 (25.3%)	90 (25.6%)	74 (24.3%)
Not living with another adult	64 (17.6%)	61 (17.3%)	50 (16.4%)
No support in pregnancy	33 (9.1%)	31 (8.8%)	25 (8.2%)
Poor/Fair/Good health	257 (70.8%)	251 (71.3%)	220 (72.4%)
Smokes	119 (32.8%)	115 (32.7%)	98 (32.2%)
Long-term illness/health problem or disability	75 (20.7%)	72 (20.5%)	61 (20.1%)
Very stressed/anxious/unhappy	104 (28.7%)	102 (29.0%)	92 (30.3%)
School < Year 12 secondary level education in Australia	203 (55.9%)	193 (54.8%)	163 (53.6%)
No person in household has paid work/earns an income	128 (35.3%)	123 (34.9%)	96 (31.6%)
Never had a job before	66 (18.2%)	63 (17.9%)	50 (16.4%)

* Percentages add to more than 100 as each participant reported multiple risks

### Data collection

Dates of families’ commencement on the program and children’s dates-of-birth were extracted from trial enrolment data [24]. At the completion of each contact with the family, the nurse or social care practitioner completed a checklist detailing the client unique identifier, date of the contact and the activities undertaken. The electronic, touchscreen enabled checklist took less than two minutes to complete. The checklist was used to record activities and content completed in the visit undertaken rather than prescribe visit content. The checklist identified activities or topics within nine domains that could be addressed during the contact: infant well-being, maternal well-being, maternal mental health, family well-being, preventive health care, environment/resources, planning and goal setting, referrals, and tools and focus modules. There were 48 items in the antenatal checklist and 56 items in the postnatal checklist.

At the visits at child ages 3, 6, 12, 26, 52 and 104 weeks the nurse completed the Session Rating Scale (SRS) with the family. The SRS is a brief subjective measure of the quality of the working alliance between the nurse and family [37]. The SRS asked the client to rate on a scale of 0–10 how well they felt understood, the degree to which the visit focused on the issues the family wanted to work on, whether the visit approach made sense and worked for the family, and whether the visit was right for them overall. The SRS was completed using the nurses’ mobile tablet and took approximately 10 minutes to complete in the context of a discussion between the nurse and client, in which they reviewed their working relationship. The SRS considers item scores less than nine to be ‘of concern’.

### Statistical methods

A visit was considered valid if it occurred in the presence of the parent (e.g. a home visit), if it occurred over the phone and covered more than five topics, or if it was a group session and covered ten or more topics. Visits were considered invalid if: there was no date of visit, no topics were covered, mother failed to attend, the visit record was duplicated, or if the visit was a phone call or group session and did not cover the minimum number of topics. Some low activity phone calls were considered valid if, based on comments recorded by the nurse, it was clear that the phone call was therapeutic in nature.

The date of each valid visit and child date-of-birth were used to determine the age of the child at the visit and then collated for each stage of the program: antenatal, and 0–6, 7–12, 13–26, 27–52, and 53–104 weeks postnatal. These data were collated at the end of the trial and descriptive statistics were used to determine family dose as compared to the program schedule, and retention in the program. Descriptive analyses were undertaken to describe the family session ratings and the proportion of visits that contained the listed activities. Exploratory factor analysis (principal components analysis) with varimax rotation was undertaken to detect components of more common activities (occurred in more than one-third of visits) that co-occurred during the program, in order to determine concordance between program aims and foci of the delivered content. The antenatal and postnatal visit data met the statistical requirements for factor analysis (antenatal KMO = 0.837, Bartlett test of sphericity p<0.001; postnatal KMO = 0.883, Bartlett test of sphericity p<0.001). An inclusive factor loading of >0.3 was chosen [38].

### Trial registration

Trial registration number: ISRCTN89962120 (21/Aug/2013), retrospectively registered.

## Compliance with ethical standards

### Ethics approval

The right@home trial was approved by the Human Research Ethics Committees in Australia of: The Royal Children’s Hospital, Victoria (HREC 32296); Peninsula Health, Victoria (HREC/13/PH/14); Ballarat Health Services, Victoria (HREC/13/BHSSJOG/9); Southern Health, Victoria (HREC 13084X); Northern Health, Victoria (HREC P03/13); and The University of Tasmania (HREC H0013113). All procedures performed in studies involving human participants were in accordance with the ethical standards of the institutional and/or national research committee and with the 1964 Helsinki declaration and its later amendments or comparable ethical standards. Written consent was obtained.

## Results

### Retention and dose

Of 363 women randomized to the intervention group, 11 (3.0%) did not commence the intervention program; 352 families commenced the intervention (97.0%) and 304 of these families completed the right@home program at child age 2 years (86.4% of program recipients; 83.7% of all trial intervention group families). The right@home program nurses delivered 9513 contacts, including 7992 valid visits. 95.3% of valid nurse visits were face-to-face home visits, ranging from 96.9% antenatal to 6 weeks postnatally to 94.3% after child-age 6 months.

Program dose was measured for families who commenced the program, that is, who received at least one or more valid nurse visits. Table 2 presents the target and expected minimum number of visits overall and for each stage of the program, together with the proportion of families receiving the minimum, median and mean total number of nurse and social care practitioners visits, and the median and mean number of nurse visits at each stage, for both those who commenced the program and for those who completed the program to child age 2 years. Families also received social care practitioner visits as needed, with the expectation that families would receive at least one.

**Table 2:** Program schedule and delivery.

	Scheduled number of visits	Target minimum (75%)	Program recipients (n = 352)			Completed Program (n = 304)		
			Received minimum N (%)	Median (Range)	Mean (SD)*	Received minimum N (%)	Median (Range)	Mean (SD)*
**Nurse visits**								
Antenatal	3	2	206 (58.5%)	2 (0 to 8)	1.9 (1.3)	182 (59.9%)	2 (0 to 6)	1.9 (1.3)
Birth to 6 weeks	5	4	310 (88.1%)	5 (0 to 9)	5.1 (1.6)	282 (92.8%)	6 (0 to 9)	5.3 (1.3)
7 to 12 weeks	3	2	309 (87.8%)	3 (0 to 7)	2.6 (1.2)	284 (93.4%)	3 (0 to 7)	2.7 (1.0)
13 to 26 weeks	4	3	289 (82.1%)	4 (0 to 11)	3.7 (1.7)	273 (89.8%)	4 (0 to 11)	3.9 (1.4)
27 to 52 weeks	4	3	291 (82.7%)	4 (0 to 15)	3.9 (2)	275 (90.5%)	4 (0 to 15)	4.3 (1.7)
53 to 104 weeks	6	4	270 (76.7%)	5 (0 to 15)	5.0 (2.8)	269 (88.5%)	6 (0 to 15)	5.7 (2.3)
Over 104 weeks	0	0	352 (100%)	0 (0 to 3)	1.0 (1.0)	304 (100%)	2 (0 to 3)	1.2 (1.0)
Total–Birth to 24 months	22	16	298 (84.7%)	23 (0 to 40)	21.3 (7.2)	287 (94.4%)	23 (0 to 40)	23.2 (5.0)
Total–Ante to 24 months	25	19	288 (81.8%)	24 (1 to 43)	23.2 (7.4)	279 (91.8%)	25 (1 to 43)	25.1 (5.2)
**Social care practitioner visits**	As required	At least 1	266 (75.6%)	1 (0 to 15)	1.7 (2.0)	239 (78.6%)	1 (0 to 15)	1.8 (2.0)
**Focus module delivery**								
					
Learning to Communicate	11	8	252 (71.6%)	9.5 (0 to 21)	9 (3.7)	242 (79.6%)	10 (0 to 21)	9.7 (3.1)
SmallTalk	5	3	15 (4.3%)	0 (0 to 4)	0.6 (0.9)	15 (4.9%)	1 (0 to 4)	0.7 (0.9)
Promoting First Relationships	11	8	267 (75.9%)	10 (0 to 24)	9.6 (4.1)	262 (86.2%)	11 (0 to 24)	10.5 (3.4)
Video feedback	6	4	211 (59.9%)	4 (0 to 12)	4.3 (2.8)	207 (68.1%)	5 (0 to 12)	4.8 (2.7)

*Mean and SD calculated only for families that received one or more visits during specified program stage

56 families did not receive any antenatal nurse visits due to recruitment occurring too late in pregnancy for a visit to be arranged, and 175 families completed the program past the child’s second birthday, up to child age 133 weeks (mean 108.4, SD 4.0). 266 (75.6%) of families received the scheduled one or more visits by the social care practitioner. 288 families received more than 75% of the scheduled nurse visits (81.8% of program families and 91.8% of completing families), and for 251 (71.3% of program families) this included one or more antenatal visits.

### Content

**Fig 1:**
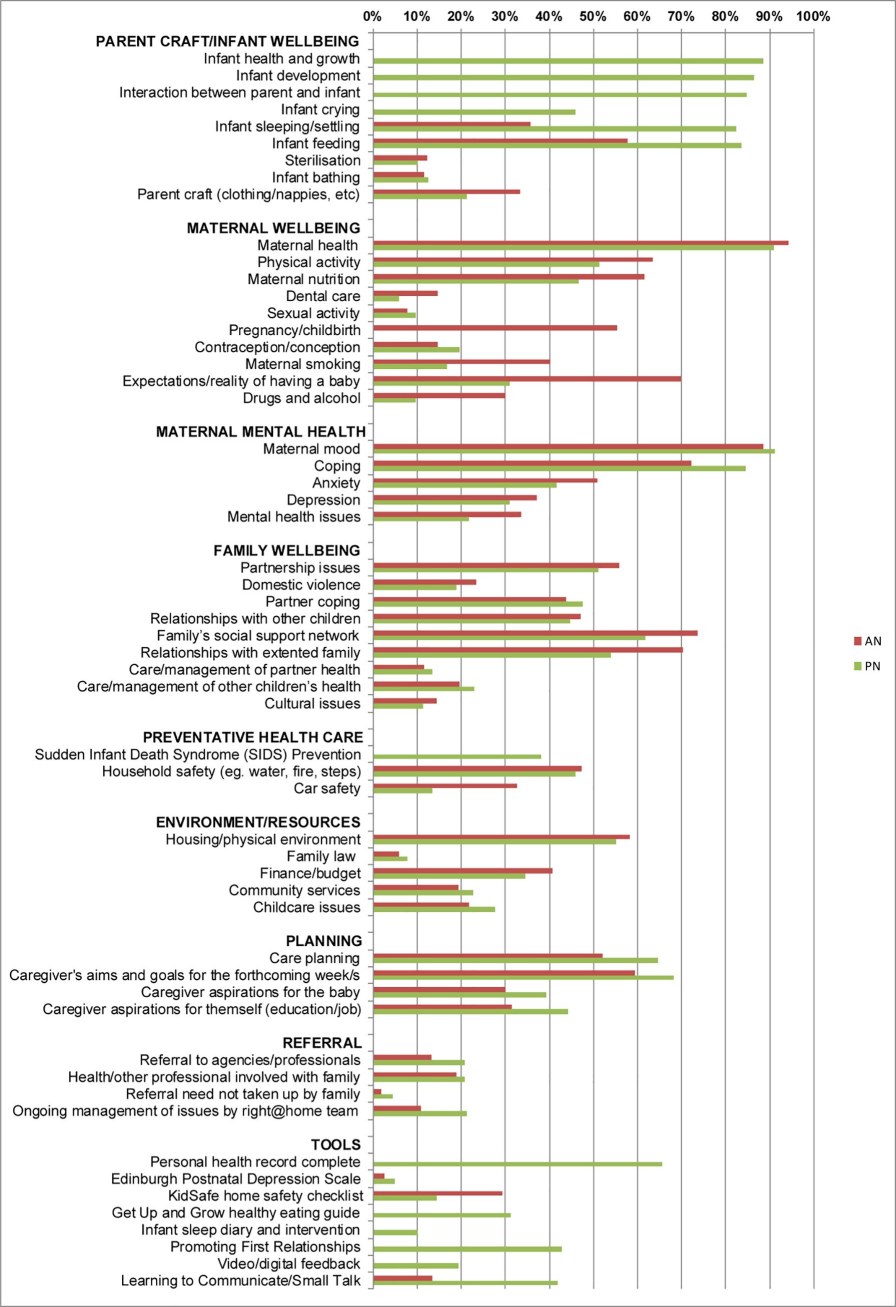
Proportion of home visits in which item was discussed. AN–Antenatal, PN–Postnatal home visit.

Fidelity was further monitored in terms of practitioner recorded delivery of program content. The proportion of valid antenatal (AN) and postnatal (PN) visits that contained each of the checklist activities is shown (Fig 1). The most common antenatal and postnatal activities were maternal health and mood, which were addressed in 94.2% and 88.6% of antenatal visits and 90.8% and 91.1% of postnatal visits respectively. The delivery of key focus module content is presented in Table 2.

As each visit contained multiple activities, factor analyses were conducted to identify which of the more common visit activities (that is, activities that were conducted in more than one-third of visits) clustered together in visits. The factor analysis resulted in six clusters for antenatal activities and six clusters for postnatal activities with eigenvalues greater than one, explaining a total of 50.3% and 47.8% of variance respectively. Table 3 presents the antenatal and postnatal visit activity components.

**Table 3:** right@home activity components and checklist activities.

Component	Variance explained (%)	Checklist activities (factor loading)
*Antenatal*		
Maternal mental health	10.4	Depression (0.848), anxiety (0.751), mental health issues (0.783), maternal coping (0.419), partnership issues (0.326).
Being prepared and healthy	10.0	Feeding (0.772), infant sleeping/settling (0.712), infant parentcraft (0.580), household safety (0.528), pregnancy/childbirth (0.430), maternal nutrition (0.406), maternal smoking (0.336).
Mother and family expectations and relationships	8.9	Relationships with extended family (0.772), families’ social support network (0.664), housing/physical environment (0.604), expectations/reality of having a baby (0.435), maternal smoking (0.450), partnership issues (0.351).
Maternal and family wellbeing	8.7	Maternal physical activity (0.669), maternal nutrition (0.575), maternal health (0.523), partner coping (0.420), maternal mood (0.404), relationships with other children (0.354), maternal coping (0.368).
Planning and goal setting	6.8	Care planning (0.785), aims and goals for forthcoming week/s (0.671), maternal coping (0.306), household safety (0.330).
Managing money	5.6	Finance/budget (0.601), and not pregnancy/childbirth (-0.549) or expectations/reality of having a baby (-0.477), maternal health (-0.358).
*Postnatal*		
Infant care and interaction	12.9	Infant development (0.813), health and growth (0.795), feeding (0.698), infant-parent interaction (0.661), sleeping/settling (0.640), completion of child health record (0.564), Promoting First Relationships (0.364).
Aspirations and planning	7.7	Caregiver aspirations for themselves (0.681), aspirations for baby (0.672), care planning (0.615), aims and goals for forthcoming week/s (0.542), finance/budget (0.395).
Maternal physical and mental health	7.5	Maternal mood (0.698), coping (0.652), health (0.584), anxiety (0.436), physical activity (0.387).
Child development, safety and maternal wellbeing	7.2	SIDS prevention (0.686), infant crying (0.544), maternal nutrition (0.475), household safety (0.386), completion of child health record (0.336), Learning to Communicate (0.305), maternal physical activity (0.305).
Family relationships and environment	6.9	Relationships with extended family (0.650), families’ social support network (0.557), housing/physical environment (0.504), household safety (0.409), maternal physical activity (0.411), partnership issues (0.344), Promoting First Relationships (0.326), and not Learning to Communicate (-0.308).
Family worries	5.6	Relationships with other children (0.574), finance/budget (0.539), partnership issues (0.516), partner coping (0.435), anxiety (0.353).

### Participant-practitioner relationship

334 participants completed the SRS at one or more of the six scheduled times: a total of 1381 SRSs were completed. The mean score for their relationship with their nurse was 39.4 (SD 1.31) out of a possible 40. There was no evidence of differences in SRS scores at any time between those who completed the program (mean 39.5 SD 1.2) and those who did not complete the program (mean 38.9 SD 1.9). 32 (2.3%) of completed SRS rated the relationship less than nine (of concern). SRS scores less than 9 were somewhat more common early in the program (SRS at 3, 6 or 12 weeks 3.0%, from 26 weeks onwards 1.5%, χ^2^_1_ = 3.6, p = 0.06), albeit the difference was not statistically significant.

## Discussion

This study explored the implementation fidelity of a SNHV program, known as right@home, for families experiencing adversity. Through systematic monitoring processes, a broad range of fidelity measures were included, covering the amount and duration of service delivery, and the quality of the relationship between the practitioner and parent/s. Unlike other fidelity reporting, extensive recording of activities conducted in the home visits allowed detailed exploration of actual content delivered and concordance with program aims. Program delivery had strong adherence to the dose, retention and scheduled focus module content, and families reported a strong working alliance with their right@home practitioner. Poor adherence to delivery of the SmallTalk program in the second year was the only area of concern.

The high retention and dose fidelity in right@home differentiates it from most other SNHV programs, with dose fidelity much higher than usually reported in home visiting research or in its real world implementation [11–13, 17]. This may be due to the lower number of 25 scheduled visits [25] when compared to other programs (for example, Nurse Family Partnership has 64 scheduled visits [12]). The intensity of visiting, however, has not been commonly noted as an issue in fidelity in the limited literature available [12–17], although qualitative research by Holland et al did suggest that the intensity of visiting in a Nurse Family Partnership program may be overwhelming for some mothers and associated with attrition [39]. It may be that the requirements of the right@home program, in terms of number of visits, was more acceptable to families than programs expecting a higher number of visits and/or more frequent visiting. In addition, the program delivery had the option to include therapeutic phone calls as ‘valid visits’. Contacts other than face-to-face home visits, although unusual, were more common after child-age 6-months, providing a flexibility that potentially allowed mothers to continue to participate and benefit from the program while accommodating work and/or school and/or a busier life. Further research is needed to understand families’ expectation of appropriate and reasonable frequency of home visiting.

The fidelity to program content and family engagement with the program may be supported by the delivery of content that was well aligned to program goals. Antenatally, the content actually delivered was consistent with the stated program aims. Similarly, postnatally, the delivered content had a strong focus on support for parenting and parenting skills. Activity components were consistent with the program aims of supporting maternal-infant bonding and attachment; mothers being future oriented and aspirational, and having enhanced coping and problem-solving skills; maternal, child and family health, development and wellbeing; positive parenting skills; and supportive relationships in their family and community. Work by Brand et al (see page 164 in [11]) and McKelvey et al [40] suggests that a content focus on parenting issues, parent-child interaction, and child development is associated with greater retention in home visiting services, more so than focusing on maternal health, life-course, relationships or case management. The postnatal content delivery of right@home was strongly focused on parentcraft and infant well-being, and may be a factor contributing to the extremely high retention in the right@home intervention.

Postnatally, the Promoting First Relationships program was an element in both the maternal-infant interaction and also family relationships components, suggesting that the program was promoting both mother-child and the child’s wider relationships, which may improve family engagement with the program [7]. Further, the child development program and relationship program were not commonly the focus of the same visit, which was not only coherent with the scheduled curriculum, but may also have ensured that each visit had a specific focus that did not overload families. The poor fidelity of delivery of the SmallTalk content, which is scheduled in the second year of the program, remains unexplained. Lack of provision of new and relevant content, particularly into the latter parts of the program, may be associated with disengagement [39]. Whilst the families in right@home did not disengage with the program overall, they may have chosen to not engage with SmallTalk materials, which were designed to reinforce and extend messages about increasing everyday use of language with children that were delivered in the Learning to Communicate content in the first year. The range of activities shows the flexibility of the program in provision of additional support in response to families’ need. Further research is needed to explore families’ perceptions of the content delivered and the ‘pacing’ of content, and the impact of these on families’ engagement with the program.

Families rated the relationship with their nurse very highly. As noted by Korfmacher (see page 465 in [19]), “services provided for free by a friendly, helpful person may be received quite positively”. Beyond the free cost and helpfulness of the right@home practitioners, use of motivational interviewing techniques; a core process component of the right@home model, is related to client engagement and retention in home visiting programs [41]. It has been suggested that programs delivered in the context of a service system with a more comprehensive approach have added value to families [7, 23]. right@home is embedded in the usual service system and includes a social care practitioner in each site’s program team. right@home program alignment with family goals, involving the wider family, clear visit focus and flexibility, and a comprehensive approach, are all elements that support family engagement in quality programs [9].

The fidelity to program retention and dose may have resulted from the timeliness and depth of the fidelity monitoring processes of external quarterly review and feedback from the MECSH Support Service team. Unlike Casillas et al. [6] who found that occasional fidelity monitoring (twice a year or less) rather than more frequent monitoring was associated with higher effects on program outcomes, our quarterly monitoring produced high fidelity. However as noted by Casillas et al. [6], it is the quality of the monitoring not frequency that matters. Albeit based on practitioner recorded data, right@home fidelity monitoring quality assurance processes were independent of the implementing services with detailed and immediate feedback to sites, which has been shown to be more likely to produce higher effect sizes in program outcomes [6].

### Limitations

Nurses and families were aware that the program was part of a trial, albeit implemented in real world service, and they consented to participate in the research. This may have increased their commitment and engagement and so may not be replicable when taken to scale. In keeping with real world service, families were not provided with incentives to participate, and the strategies for supporting quality were those used in usual MECSH implementation suggesting that with appropriate technical, training and fidelity support, the quality should be replicable.

In addition, the primary measure of the quality of the relationship between the nurse and the family, the SRS [37], is a subjective measure completed in collaboration between the nurse and the family, and may be subject to social desirability bias. The SRS does, however, recognise this and assigns a high cut-off score of 9 out of 10, and the positive changes in the scores over time suggests that the quality of the relationships were good and improved as the nurse-family relationship developed.

## Conclusions

The right@home program was implemented with fidelity. Families in the right@home trial intervention group were retained in the program for the expected duration, and received the program dose as scheduled in terms of the total number of visits overall and in each program stage. Antenatal and postnatal content actually delivered was consistent with the program aims. The retention of families in the program and the proportion of schedule dose received were much higher than achieved in comparable home visiting programs. The families also rated the relationship with their nurse provider consistently highly. This fidelity was particularly notable as the right@home program was delivered through usual child and family health services, rather than using nurses or systems especially established for the research. The structure and flexibility of the program and alignment with families’ capacity, together with the timeliness and depth of the quality monitoring processes, may have contributed to program fidelity that was superior to most previously evaluated home visiting programs.

## Supporting information

S1 File Reporting guidelines_CARE_table. (DOCX)
