# Supplementary material for: Quality of delivery of “right@home”: Implementation evaluation of an Australian sustained nurse home visiting intervention to improve parenting and the home learning environment
Source: PLoS One. 2019 May 6;14(5):e0215371. doi: 10.1371/journal.pone.0215371 (PMC6502332; doi:10.1371/journal.pone.0215371)
Supplement: S1 File — (DOCX) [file pone.0215371.s001.docx]

Table 1. Reporting guidelines for implementation research of nurturing care interventions designed to promote early child development

| C.A.R.E. (consolidated advice for reporting ECD implementation research) guidelines | | | |
| --- | --- | --- | --- |
| Section of report | Item number | Item label | Article Compliance |
| **Introduction** | 1 | Previous evidence about the  intervention | Introduction paragraph 1 |
|  | 2 | Effectiveness of the present  intervention (if known) | Not yet known/published |
|  | 3 | Rationale for the  implementation research | Introduction paragraph 2 |
|  | 4 | Aims and objectives of the  implementation research | Introduction final paragraph |
| **Methods** | 5 | Context of implementation  for the intervention | Method: Study design and setting paragraph 1 |
|  | 6 | Implementation strategy for  the intervention | Method: Study design and setting paragraph 2 |
|  | 7 | Implementation recipients for  the intervention | Method: Participants and Table 1 |
|  | 8 | Intended intervention content | Method; Study design and setting paragraph 2 |
|  | 9 | Changes to intervention  content | Not applicable |
|  | 10 | Intended intensity/total  exposure to the  intervention | Included in Results Table 2 |
|  | 11 | Personnel involved in  supporting the  implementation of the  intervention  (e.g., coordinators,  trainers, supervisors, and  ancillary staff) | Method; Study design and setting paragraph 3 |
|  | 12 | Personnel delivering the  intervention (e.g., mother  leaders, community health  workers, and teachers) | Method; Study design and setting paragraph 2 |
|  | 13 | Methods to assess fidelity  regarding delivery of  intervention | Method; Study design and setting paragraph 3 |
|  | 14 | Methods to assess  understanding and  enactment of intervention  skills by recipients | Method; Study design and setting paragraph 3 |
|  | 15 | Implementation research data  collection team | Method: Data collection |
|  | 16 | Sampling and data  management procedures | Method: Statistical methods paragraph 1 |
|  | 17 | Plan of analysis for  implementation data | Method: Statistical methods paragraph 2 |
| **Results** | 18 | Results of the implementation  Evaluation | Results |
| **Discussion** | 19 | Interpretation of findings of  the implementation  evaluation | Discussion |
|  | 20 | Strengths and limitations of  the implementation  research | Discussion: Limitations |
|  | 21 | Scalability and sustainability  of the intervention and  implementation strategy | Not included in this study: discussion regarding scalability included in LImitations |
